# Treatment of Wastewater Containing Nonsteroidal Anti-Inflammatory Drugs Using Activated Carbon Material

**DOI:** 10.3390/ma15020559

**Published:** 2022-01-12

**Authors:** Florinela Pirvu, Cristina Ileana Covaliu-Mierlă, Iuliana Paun, Gigel Paraschiv, Vasile Iancu

**Affiliations:** 1 Faculty of Biotechnical Systems Engineering, Politehnica University of Bucharest, 060042 Bucharest, Romania; florinela_pirvu@yahoo.com (F.P.); paraschiv2005@yahoo.com (G.P.); 2 National Research and Development Institute for Industrial Ecology—ECOIND, 060652 Bucharest, Romania; iuliana_paunita@yahoo.com (I.P.); vasileiancu@yahoo.com (V.I.)

**Keywords:** wastewater treatment, activated carbon, anti-inflammatory drugs, ibuprofen, acetaminophen, diclofenac, ketoprofen

## Abstract

This study presents an adsorbent material (activated carbon) used in the treatment of wastewater with the role of removing ibuprofen, acetaminophen, diclofenac and ketoprofen pollutants. The wastewater treatment efficiencies of the activated carbon were systematically investigated using adsorption kinetics. The parameters studied were: pH (4 and 6 values of pH), initial concentration of wastewater (1, 5, and 10 mg/L), contact time (10 min), adsorbent quantity (0.1, 0.5, and 1 g), and isotherm models (Langmuir and Freundlich). The highest wastewater treatment efficiency was obtained at the 6 pH value. The determination of four anti-inflammatory drugs, frequently monitored in wastewater, was performed by a simple and fast method using the HPLC-technique-type DAD (diode array detector). The method was linear when the concentration ranged between 0.5 and 20 m/L for all compounds. The equilibrium concentration was obtained after 8 min. The octanol/water coefficient influenced the removal efficiency of the four drugs by the adsorbent material (activated carbon). The dose of activated carbon (0.1 to 1 g) significantly influenced the efficiency of wastewater treatment, which increased considerably when the dose of the adsorbent material increased. Using 1 g of the adsorbent material for the treatment of wastewater containing 1 mg/L initial concentration of pollutant compounds, the efficiencies were 98% for acetaminophen, 92% for diclofenac, 88% for ketoprofen and 96% for ibuprofen.

## 1. Introduction

The factors that led to the emergence of pharmaceuticals in the environment are: industrialization, urban development, irrational drug use, and inadequate waste management policies. The effects of water pollution with drugs and their residues include aquatic toxicity, development of resistance to pathogenic bacteria, genotoxicity, and endocrine disorders and endanger human health. There are various correlations between the environment and human health affected by drug residues: environmental xenobiotics influence male and female fertility due to long-term mutagenic effects [1,2].

The immune system is especially vulnerable to the harmful effects of xenobiotics, and immunotoxicity can lead to low resistance to infections, tumor production, or an increased incidence of autoimmune diseases.

The definition of a drug starts from the fact that it is a xenobiotic because it comes from an external source (xenon) and is active in a biological unit (biotic) [3], and it can be obtained from a natural or synthetic/semisynthetic source, having the ability to control biochemical defects.

Typically, there are a variety of therapeutic classes that are used in both human and veterinary medicine to treat or prevent disease [4].

The literature presents drugs and drug residues in both hydrosphere, soil and biota [5].

Organic micropollutants are produced due to uncontrolled disposal in industrial wastewater effluents, hospital effluents, and municipal septic tank effluents and growth promoters in agriculture, which may threaten public health and have led to worldwide toxicological concerns [6,7,8]. The performance of activated carbon powder was evaluated in another study of wastewater treatment containing some environmental pollutants (organic and inorganic) [8].

There are currently international toxicological and epidemiological databases containing limits for daily doses and tolerable doses of drugs and drug residues in drinking water, as well as various assessments of the chemical risk of exposure to these substances [9].

Drug contamination of drinking water can be indirectly caused by effluents from wastewater treatment plants, which are the main carriers of pharmaceuticals and their metabolites in receiving water sources, such as rivers, lakes, and groundwater aquifers, which are used for obtaining drinking water [9]. Pharmaceutical products are present at the trace level in drinking water; even in this case, the question about the efficiency of wastewater treatment in treatment plants is asked.

The factors that influence the treatment efficiency of wastewater with drug content depend on the structure of the drug, the temperature of the treatment process, the degree of hydrophobicity, and the retention time of the drug in the environment [9,10,11,12,13,14].

Below are some data from the literature on the concentration of drugs and the maximum limits found in the environment according to Table 1 and the WHO [15] (Table 2).

As can be seen from Table 1, the concentration of pollutants expressed in ng/L is very low.

At the national level, there are no limits on the concentration of the four drugs in drinking water or wastewater. Based on these aspects, this study was carried out because in the future it is desired to implement an infrastructure and a plan for monitoring these pollutants in drinking water.

Due to the large number of pharmaceuticals and different physicochemical properties, pharmaceutical residues are often inefficiently removed from conventional wastewater treatment. In the literature is mentioned the removal of acetaminophen, diclofenac, ibuprofen, and ketoprofen using activated carbon as adsorbent material [9,10,11,12,13,14].

Pharmaceuticals end up in the environment in the form of residues in rivers and sewage effluents and then in surface waters, such as soil and drinking water. This adsorption method can be economically and technically favorable for wastewater treatment. The technique consists in the ability of the adsorbent material to absorb on its surface the pharmaceutical residues from the wastewater. The specific surface area of the adsorbent material may be important because the removal efficiency may increase with increasing surface area [14,20,21].

The study consists of the development of a simple, fast, accurate, and precise method for the simultaneous quantitative determination of acetaminophen, ketoprofen, ibuprofen, and diclofenac in wastewater using the HPLC technique and studies of their removal from wastewater using an adsorbent on activated carbon [13,20,21,22].

## 2. Materials and Methods

### Chemicals and Reagents

The materials were purchased from Sigma-Aldrich (Burlington, MA, United States). The purity of ibuprofen was ≥97.0%, acetaminophen ≥95.0%, diclofenac ≥ 99.0%, and ketoprofen ≥ 99.0%. The solvents used for liquid chromatography analysis, acetonitrile and methanol, were purchased from Honeywell, and ammonium acetate was obtained from Sigma-Aldrich. The activated carbon was purchased from Trace Elemental Instruments with a particle size between 10 and 50 µm. The adsorbent material (activated carbon) presents a specific surface area of 256 m^2^/g, a pore size of 12.7 Å, and a total pore area of 870 m^2^/g. The chemicals and reagents present analytical purity specific to the methods described in this study.

The molecular structure and some physicochemical properties of drugs are presented in Table 3. The factors that are influencing the removal efficiency of environmental pollutants are given by the hydrophobicity of the drug, size and molecular structure, solubility, and dissociation constant (pKa) [23,24].

## 3. Results and Discussion

### 3.1. Analytical Method (HPLC (High Performance Liquid Chromatography))

**Figure 1:**
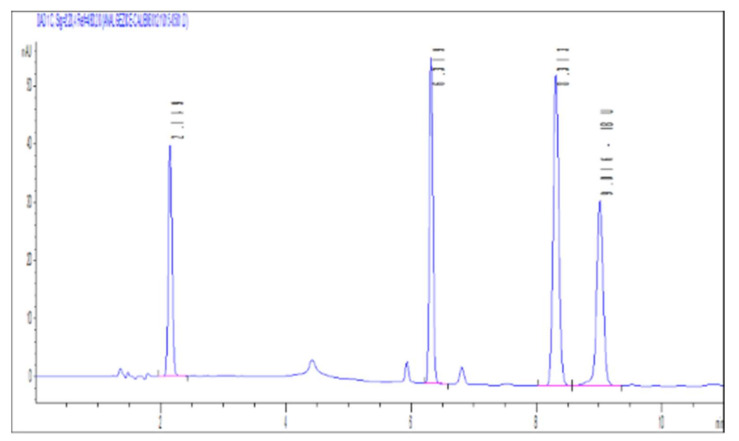
Chromatogram obtained by analyzing a mixed solution of ACF, KTF, DCF, and IBF at 4 wavelengths corresponding to the absorption maximum.

All the studies were made using synthetic wastewater. For the analysis and quantification of the four drugs studied, the HPLC method was used, whose optimal separation parameters are: Eclipse C18 chromatographic column with a size of 4.6 mm × 150 mm and a particle size of 5 μm; mobile phase: 20 mM phosphate buffer in ultrapure water (pH = 3.3): acetonitrile with gradient elution; injection volume of 10 μL and flow rate of 1 mL/min; UV detection: λ = 248 nm for acetaminophen and 255 nm for ketoprofen, and for diclofenac and ibuprofen, absorption maxima at 280 nm and 220 nm, respectively; separation time: 10 min (Figure 1).

The order of separation of the compounds on column C18 is as follows: acetaminophen > ketoprofen > diclofenac > ibuprofen.

The calibration curves of the four drugs were performed in the linear range between 0.5 and 20 µg/L, with correlation coefficient values (R^2^) higher than 0.999 for each of the four anti-inflammatory drugs studied. All the methods developed in this study were validated internally. The parameters that were evaluated were linearity, accuracy, precision, detection limit (LOD), and quantification limit (LOQ) according to references [25,26].

#### 3.1.1. Linearity

To determine linearity, five working solutions were prepared for all drugs studied in the 0.5–20 µg/L measuring range. The parameters of linearity showed that the intercept values were small and the correlation coefficient was close to one for all the studied compounds (Table 4).

The equation of the linear regression function corresponding to the calibration curve for each analyte has a linear dependence of the values of the area of the chromatographic peaks on its concentration. The concentration range on which the detector response is proportional to the concentration of the compounds is between 0.5 and 20 μg/L for the four analytes studied.

#### 3.1.2. The Accuracy of the Analytical Method

The accuracy of the analytical method shows to what extent the value determined for an analyte in a sample corresponds to the true value. This represents the systematic deviation of the measured results from the true result. The accuracy of the method is also an indicator of the usefulness and applicability of this method to real evidence [24].

The four compounds (ibuprofen, paracetamol, ketoprofen, and diclofenac) were quantified by HPLC method. The recovery yield refers to the ratio between the experimentally determined concentration (obtained) using the interpolation on the calibration curve of each analyte and the concentration added in the aqueous matrix [24]. In order to determine the recovery efficiency, the synthetic wastewater samples were enriched with a known concentration of the four studied compounds of about 10 µg/L (Table 5).

The BIAS of the method was demonstrated from the recovery yields of the four compounds ranging from 81% to 96% (Table 5).

#### 3.1.3. The Precision of the Analytical Method

The precision of an analytical method expresses the fit or degree of agreement between a series of determinations obtained from several samples from the same homogeneous sample under specific conditions. The precision can be assessed at three levels: repeatability, intermediate precision, and reproducibility.

The precision of the entire analytical procedure, expressed as a relative standard deviation (RSD%), was determined by repeated analysis of real samples of wastewater from a wastewater treatment plant.

To determine the repeatability, the actual 5 µg/L concentration samples were read six times in a single day; for reproducibility, the 5 µg/L concentration samples were read four times for 3 consecutive days. The precision of the method varied in the case of repeated measurements below 10% (Table 6).

### 3.2. Adsorption Studies

Adsorption experiments were performed in 100 mL conical flasks. Stock solutions were prepared in methanol, and subsequent dilutions were performed using wastewater matrices as sample diluent. Volumes of 50 mL of different concentrations (1, 5, and 10 mg/L) of each studied anti-inflammatory were contacted with 0.1, 0.5, and 1 g of activated carbon and stirred on an orbital shaker at 250 rotations. After each experiment, the supernatant was filtered and subjected to HPLC analysis at different wavelengths corresponding to the analytes of interest (acetaminophen at 248 nm, diclofenac at 280 nm, ketoprofen at 255 nm, and ibuprofen at 220 nm). The mathematical models applied to characterize the adsorption processes are described in Table 7.

**Figure 2:**
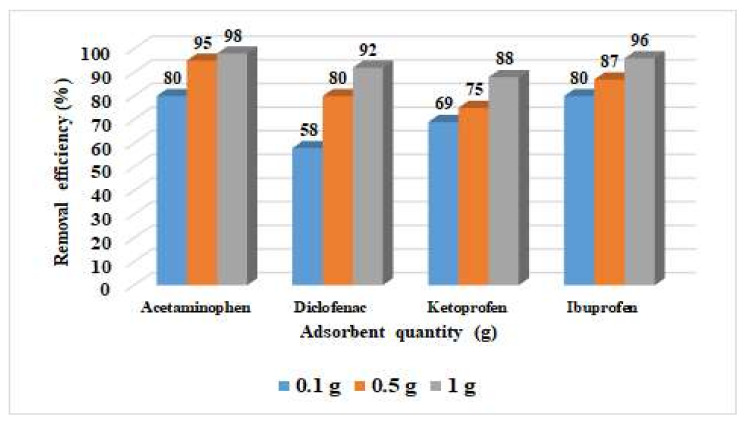
Removal efficiency versus adsorbent quantity.

The removal efficiency increased considerably when the amount of adsorbent material was increased from 0.1 to 1 g of activated carbon. The highest adsorption efficiencies of 98% for acetaminophen and 92% for diclofenac were achieved at 1 mg/L for each pharmaceutical compound and 1 g of adsorbent material. The highest removal efficiencies, 88% for ketoprofen and 96% for ibuprofen, were obtained considering the experiments conducted using 1 mg/L of ketoprofen/ibuprofen and 1 g of activated carbon (Figure 2).

**Figure 3:**
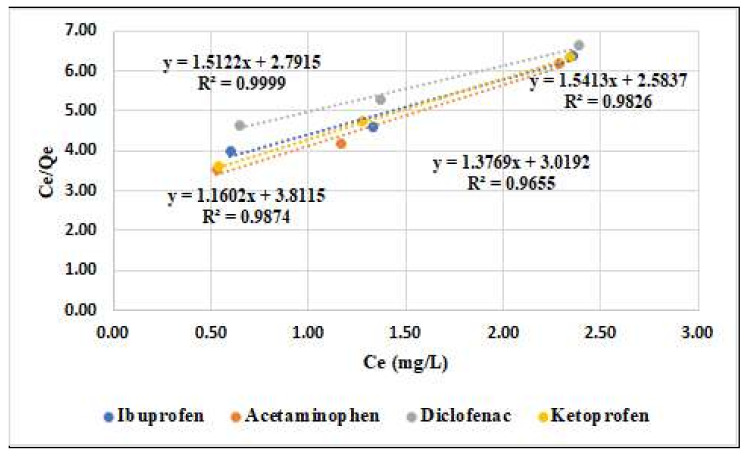
Langmuir linearized isotherm for ibuprofen, acetaminophen, diclofenac, and ketoprofen onto activated carbon.

The linear profile of the Langmuir isotherm was obtained by graphical representation of the Ce/Qe ratio versus Ce in Figure 3.

**Figure 4:**
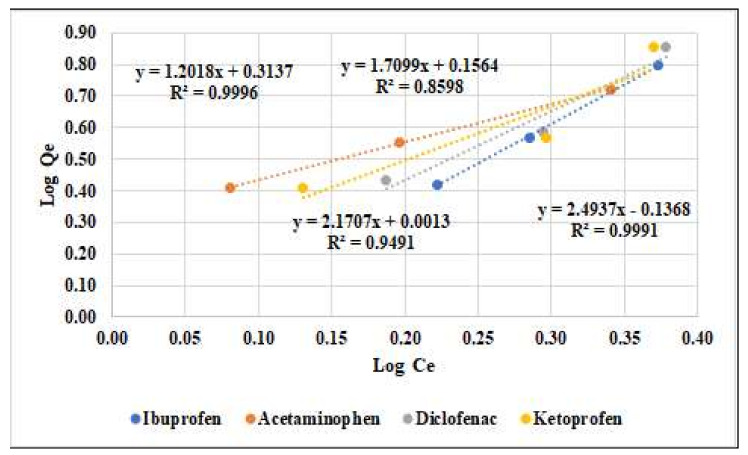
Freundlich linearized isotherm for ibuprofen, acetaminophen, diclofenac, and ketoprofen onto activated carbon.

The linear profile of the Freundlich isotherm was obtained by graphical representation of the log Q versus log Ce in Figure 4.

The comparison of the coefficient of correlation (R^2^) indicates that the Langmuir isotherm fits better results (R^2^ = 0.9655 to 0.9999) compared with the Freundlich isotherm (R^2^ = 0.8598 to 0.9996) for the adsorption of four compounds onto active carbon.

The Langmuir constant R_L_ is in the range of 0–1, indicating that the retention of compounds is favorable as it can be seen in Table 8. The adsorption of four compounds onto the activated carbon is favorable for values of R_L_ (Langmuir constant) of 0.1 < 1/n < 1.0.

Table 9 and Figure 3 and Figure 4 reveal that the Langmuir model (correlation coefficient R^2^ = 0.9655 to 0.999, respectively) better describes the adsorption isothermal behaviors for the compounds studied onto the activated carbon instead of the Freundlich model.

In this study, we evaluated parameters: the solution pH (4 and 6), contact time (10 min), and initial concentration (1, 5, and 10 mg/L) of the four compounds’ solution and adsorption isotherms (Langmuir and Freundlich). The equilibrium concentration was obtained after 8 min at the 6 units of pH for all compounds. When the concentration increased (1 to 10 mg/L), the efficiency removal decreased.

### 3.3. Desorption Study

**Figure 5:**
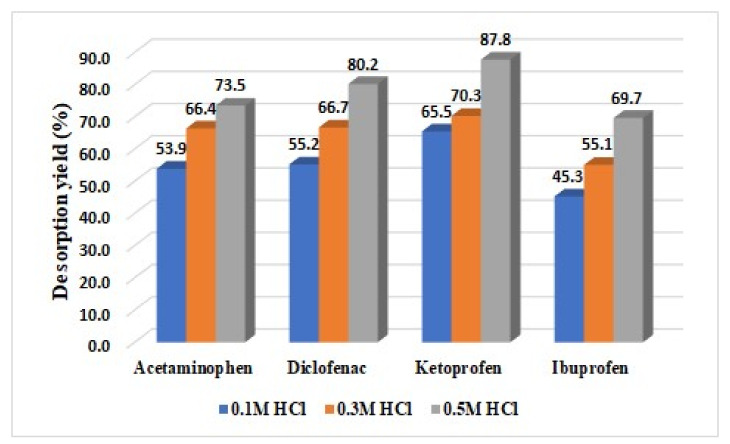
Desorption yields of drugs.

The same procedure was applied for the desorption study as in the case of adsorption studies, with the exception that an acid solution (hydrochloric acid of different concentrations (0.1, 0.3, and 0.5 M)) was used instead of the aqueous solution with drugs for the removal of the drugs retained on activated carbon. The HPLC technique was used for the quantification of the pollutants’ concentration. The desorption study results are presented in Figure 5.

The desorption yields of the drugs retained on the activated carbon surface were influenced by the concentration of hydrochloric acid used, as can be seen in Figure 5. The order of desorption is not the same as the order of adsorption, as can be seen in Figure 5: 87.8% ketoprofen > 80.2% diclofenac > 73.5% acetaminophen > 69.7% ibuprofen due to dissociation constant and solubility.

## 4. Conclusions

The batch adsorption study of ibuprofen, acetaminophen, diclofenac, and ketoprofen from wastewater was carried out by using activated carbon as adsorbent material. Different isotherms, such as Langmuir and Freundlich, were studied at three different pH values (4 and 6), and it was found that the adsorption characteristics were well predicted by the Langmuir adsorption isotherm. The Langmuir best results are explained by the correlation coefficient, wherein R^2^ is higher than Freundlich (R_L_^2 ^ > R_F_^2^). The equilibrium parameter (R_L_) for the Langmuir isotherm was in the range of 0–1, which indicates that the adsorption process is favorable for all the compounds studied. The state of equilibrium for all the compounds in adsorption studies was achieved after 8 min. From the studies of adsorption onto activated carbon material, it can be concluded that the highest wastewater treatment efficiencies are in the following order: 98% acetaminophen > 96% ibuprofen > 92% diclofenac > 88% ketoprofen. The desorption studies depend on the dissociation constant and solubility of the compounds: 87.8% ketoprofen > 80.2% diclofenac > 73.5% acetaminophen > 69.7% ibuprofen.

This study achieved its desired goal by developing and implementing the HPLC methods for the ibuprofen, acetaminophen, diclofenac, and ketoprofen compounds of interest, as well as by removing these compounds from wastewater using activated carbon. The pharmaceutical compounds and metabolite residues coming from pharmaceutical products for human and veterinary use can be removed from wastewater using activated carbon as adsorbent material.

## Figures and Tables

**Table 1:** Concentrations of diclofenac, ibuprofen, and acetaminophen in the environment.

Compound	Effluent (Maximum Concentration, ng/L)	Rivers Waters (Maximum Concentration, ng/L)	References
Diclofenac	2349	568	[16]
	598	<LOQ	[17]
Ibuprofen	27,256	5044	[16]
	4239	2370	[17]
Acetaminophen	<20	-	[17]
	-	555	[18]

“<LOQ”—limit of quantification.

**Table 2:** Concentrations of diclofenac and ibuprofen found in surface waters [15].

Compound	Austria Maximum Conc. (ng/L)	Finland Maximum Conc. (ng/L)	France Maximum Conc. (ng/L)	Germany Maximum Conc. (ng/L)	Ref.
Diclofenac	64	40	41	1200	[19]
Ibuprofen	nd	65	120	530	[19]

“nd”—not detected; “Conc. “—concentration; “Ref. “—references.

**Table 3:** Drugs’ physicochemical properties.

Drugs	Structural Formula	Molecular Formula	Molecular Mass (g/mol)	Log Kow
Ibuprofen (IBF)	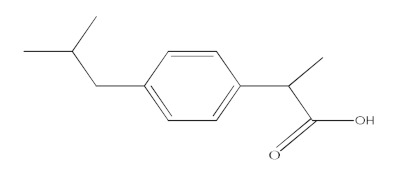	C_13_H_18_O_2_	206.13	3.97
Ketoprofen (KTF)	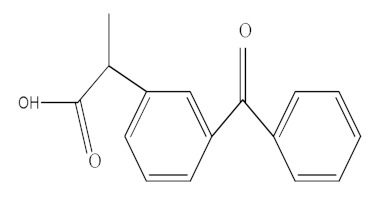	C_16_H_14_O_3_	254.09	3.12
Diclofenac (DCF)	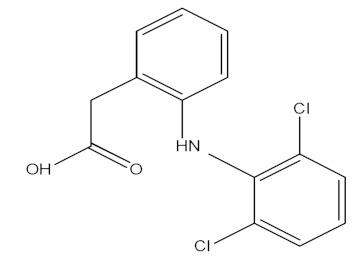	C_14_H_11_Cl_2_NO_2_	295.02	4.51
Acetaminophen (ACF)	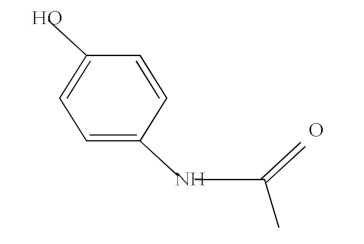	C_8_H_9_NO_2_	151.06	0.46

**Table 4:** Linear regression parameters.

Analyte	Regression Equation	R^2^	LOD (µg/L)	LOQ (µg/L)
Acetaminophen	y = 38.94x + 11.68	0.9991	0.10	0.30
Ketoprofen	y = 33.13x + 0.26	1.0000	0.20	0.60
Diclofenac	y = 17.03x + 3.89	0.9997	0.10	0.65
Ibuprofen	y = 21.75x + 8.98	0.9991	0.03	0.15

**Table 5:** The accuracy of the analytical method.

Analyte	Added Concentration (µg/L)	Obtained Concentration (µg/L)	Recovery (%)
Acetaminophen	10	9.599	95.99
Diclofenac	10	8.145	81.45
Ketoprofen	10	8.392	83.92
Ibuprofen	10	9.022	90.22

**Table 6:** Precision data obtained on real sample.

Analyte	Concentration (µg/L)	Repeatability (RSD %) (n = 6)	Reproducibility (RSD %) (n = 12)
Acetaminophen	5	0.15	0.29
Ketoprofen	5	0.11	0.28
Ibuprofen	5	0.29	0.40
Diclofenac	5	0.17	0.23

”n”—number of experiments.

**Table 7:** Mathematical models.

Mathematical Model	Equation	References
Langmuir	$\frac{C_{e}}{Q_{e}}=\frac{1}{Q_{m\;}\times K_{L}}+\frac{C_{e}}{Q_{m}}$ equilibrium parameter (R_L_) $R_{L}=\frac{1}{1+K_{L}\times C_{0}}$	[21]
Freundlich	$\log Q_{e}=\log K_{F\;}+\frac{1}{n}\times logC_{e}$	[21]

**Table 8:** R_L_ value of the metals in the Langmuir isotherm.

Compound	Initial Concentration (mg/L)		
	1	5	10
Acetaminophen	0.6623	0.2817	0.1639
Diclofenac	0.0132	0.0027	0.0013
Ketoprofen	0.7737	0.4061	0.2548
Ibuprofen	0.5000	0.1667	0.0909

**Table 9:** Langmuir and Freundlich adsorption parameters.

Adsorbent Material	Langmuir Parameters			Freundlich Parameters		
	Q_max_ (mg/g)	K_L_ (L/g)	R^2^	K_F_ (m/g)	1/n	R^2^
Ibuprofen	0.70	2.07	0.9655	2.49	0.14	0.9991
Acetaminophen	0.64	1.62	0.9826	1.20	0.31	0.9996
Diclofenac	0.85	3.23	0.9874	1.55	0.21	0.9491
Ketoprofen	0.66	1.85	0.9999	1.71	0.16	0.8598

## Data Availability

Not applicable.
